# Exploring the Relationship of Image Formation on Tourist Satisfaction and Loyalty: Evidence From China

**DOI:** 10.3389/fpsyg.2021.748534

**Published:** 2021-11-23

**Authors:** Abdelhamid Jebbouri, Heqing Zhang, Lei Wang, Nasser Bouchiba

**Affiliations:** ^1^Department of Tourism Management, Guangzhou University, Guangzhou, China; ^2^Faculty of Hospitality and Tourism, Xuzhou University of Technology, Xuzhou, China; ^3^Department of Political Science, Sun Yat-sen University, Guangzhou, China

**Keywords:** Kaiping cultural heritage destination, local community participation, authenticity, access to local products, tourist satisfaction, tourist loyalty

## Abstract

Cultural heritage tourist destinations have emerged as a hot topic in tourism literature, but there have been relatively few studies that determine the role of the involvement of local participation in either tourism planning or the decision-making processes of tourists. The connection between local community participation (LCP), authenticity, access to local products, destination visit image, tourist satisfaction, and tourist loyalty is thus relatively unexplored in the literature. This study used a quantitative approach based on a survey with 406 respondents visiting the city of Kaiping in Guangdong, China. The proposed hypotheses were empirically tested with SPSS and Analysis of Moment Structures. The resulting outcomes indicated a positive correlation between LCP, authenticity, and access to local and destination visit image, which led to tourist satisfaction and ultimately resulted in tourist loyalty. Additional theoretical contributions, practical implications, and limitations were also discussed.

## Introduction

Tourism is when people travel from one place to another to stay or work and spend temporary leisure time at their destination (Towoliu et al., 2020). Tourism can be categorized by the motives of the tourists in the destinations. The motivation for attracting tourism may result from different cultural endorsements, natural reserves, and maritime features (Song et al., 2020). The socio-cultural attributes of a country provide added value for the tourism industry (Moscardo, 2015). Cultural heritage tourism destinations are gaining popularity, and culture has been identified as a significant predictor of intention to visit (Lee et al., 2020).

China is well-endowed in terms of cultural heritage, with centuries of historical background (Song et al., 2020). In 1986, the Chinese government designated tourism as a broad economic activity with the explicit goal of launching the modernization program of the country. Tourism was designated as part of the national plan of China for social and economic development for the first time (Sharpley and Telfer, 2015).

In contrast to prior political emphasis, the government of China has made tourism growth a priority, emphasizing the economic benefits of the sector. In 2002, the Chinese government defined historical spots as areas endowed with an exceptional abundance of cultural artifacts of substantial historical significance or high revolutionary memory significance (Li, 2002). In 2003, China was granted protection under the UNESCO International Convention for the Preservation of Intangible Cultural Heritage (Yang et al., 2019). Meanwhile, the Chinese government maintained its focus on recognizing new cultural areas through legislation (Song et al., 2020).

The development of tourist destinations in China may have economic and socio-cultural effects on the local communities. These effects may benefit the communities or act as a barrier to accepting tourism (Song et al., 2020). Apart from economic benefits, local communities are also attracted to the commoditization of cultures from minority ethnic groups propagated through ethnic tourism (Towoliu et al., 2020). Such cultural elements attach enormous spiritual and symbolic importance to their art objects and ceremonies, which may help attract tourists. For instance, Tibetan traditional funeral ceremonies have been considered a tourist attraction (Towoliu et al., 2020).

Most empirical studies have proposed destination familiarity as a single-dimension construct measured through visiting experiences from previous destinations (Stylidis, 2020) that has a positive correlation to destination image (Chi et al., 2020). These studies have indicated that familiarity can be used to explain the differences in the various aspects of travel behavior among regular and first-time visitors (Chi et al., 2020). Researchers have argued that familiarity contributes greatly to the destination choice and behavioral intentions of visitors (Kim et al., 2019; Chi et al., 2020). However, recent studies have shown that familiarity with a tourist destination can be conceptualized as having two dimensions: experiential and informational (Chen and Lin, 2012; Kim et al., 2019). Kim et al. (2019) indicated that most previous studies on destination familiarity have measured experiential familiarity and thus failed to consider the indirect familiarity of consumers (without consumption experience) with a destination, which needs to be incorporated into the measurement of destination familiarity (Kim et al., 2019). This could include, for example, the geographic distance between the origin of the tourist and the destination country (Stylidis, 2020), personal contact with others (Terzidou et al., 2018), awareness of service quality (Chi et al., 2020), and destination information acquisition (Kim et al., 2019), among others. However, despite the potential significance of destination familiarity for destination marketing, relatively limited research has addressed the extent to which different aspects of familiarity affect the perceptions and intentions of visitors concerning a tourist destination (Kim et al., 2019).

The sustainability of the tourism industry is inextricably linked to the many economic, sociocultural, and political climates that exist within the host community (Thetsane, 2019). Local participation, in particular, has been viewed positively as a catalyst for change and authorization for the progress of a country. It is generally thought that residents are eager and able to engage equally in the management, decision-making, and managing of tourist development (Stone and Stone, 2011). More importantly, local community involvement can strengthen the character of most tourism destinations (Chili and Ngxongo, 2017). Unfortunately, most previous studies have only investigated the role of the local communities in the overall tourism planning and development process (Michael, 2009; Kim et al., 2013), while very few studies have been conducted to discover the grounds for such involvement by communities in the decision-making of tourists (Chili and Ngxongo, 2017), particularly characterized in developing countries (Chili and Ngxongo, 2017; Thetsane, 2019). The findings on the influence of the role of local communities on the destination choice of tourists are limited, resulting in the lack of a standardized definition and coherent foundation for research. Therefore, this study extends the knowledge related to the role of local communities in shaping the destination visit image (DVI) and, more specifically, how the participation of local communities, destination authenticity, and access to local products influence DVI and tourist satisfaction, further leading to tourist loyalty toward cultural heritage sites.

Based on the discussion above, this study proposed three research questions:

RQ1: How do local community participation (LCP), authenticity, and access to local products influence the formation of tourist DVI?

RQ2: How does DVI influence tourist satisfaction and tourist loyalty?

RQ3: How does tourist satisfaction influence loyalty toward cultural heritage tourism?

This manuscript is organized as follows. In section “Introduction,” we presented the literature review. Section “Theoretical Background and Hypothesis Development” presents the development of the hypotheses, while section “Materials and Methods” provides the research methods, and section “Data Analysis and Results” explains the statistical analysis of this study. Section “Discussion and Conclusion” presents the discussion and concluding remarks. Finally, the last part of this study presents the limitations and directions for future research.

## Theoretical Background and Hypothesis Development

### Destination Familiarity

Product or brand familiarity has frequently been linked to several past product-related experiences that consumers have accumulated (Stylidis, 2020), and it is one of the most important variables in the marketing literature (Kim et al., 2019). Within the literature on tourism, destination familiarity has always been treated as a single-dimension construct measured through previous destination experience (Hu and Ritchie, 1993), which can be used to contrast first visits and revisits (Prentice, 2006). In other words, destination familiarity is the basis for explaining differences in various aspects of travel behavior between the images of a given tourist destination of visitors and non-visitors (Andreu et al., 2000). Researchers have reported that many visitors feel secure in a familiar destination, while novel destinations are likely to seem riskier to visitors (Lepp and Gibson, 2003). Therefore, destination familiarity allows researchers to understand how visitors shape their mental image of a particular destination (Chen and Lin, 2012).

Nevertheless, researchers have criticized the measurement of the single-dimension construct of destination familiarity, noting that is not appropriate in the temporal tourism environment (Stylidis, 2020; Ju et al., 2021). According to Kim et al. (2019), destination familiarity can be increased by direct experiences, such as purchasing and usage behavior, while indirect experience can also significantly influence the familiarity of a tourist with a given destination; this can include exposure to advertisements, reviews from others, the geographic distance between origin and destination, having received information regarding a given destination, perceived knowledge related to education, media coverage, travel guides, social media, and personal contact with others (Kim et al., 2019; Chi et al., 2020; Stylidis, 2020; Ju et al., 2021). Researchers have therefore suggested that two aspects of destination familiarity should be operationalized: experiential and informational (Kim et al., 2019). Experiential destination familiarity refers to the level of awareness created through past travel experience, whereas informational familiarity is accumulated by indirect exposure to a destination (Baloglu, 2001). The results of many studies have demonstrated that destination familiarity based on past travel experience significantly affects the travel decision-making process of visitors (Prentice, 2006; Tan and Wu, 2016), but visitors can still attain a definite level of destination familiarity *via* indirect experience (Chi et al., 2020). Other means of direct experience in creating destination familiarity have largely been overlooked in tourism literature (Ju et al., 2021).

### Destination Visit Image Formation

The formation of DVI is among the factors that play a significant role in creating tourist loyalty (Králiková et al., 2020). The importance of this concept has led to a growing body of studies on tourism destinations (Alcocer and Ruiz, 2019), and the findings of these studies indicate that DVI has a significant impact on loyalty, satisfaction (Ragab et al., 2019), visit intention (Wang et al., 2021a), and revisit intention (Melo et al., 2017). However, there is currently no agreement on a precise definition for this concept (Ragab et al., 2019) because previous studies have defined DVI in many different ways. Prior studies defined DVI by incorporating a set of feelings, emotions, and attitudes that tourists hold regarding the destination (Ragab et al., 2019). The most dominant definition of DVI in previous studies is the overall impression of tourists on a specific destination based on their knowledge and feelings about that destination (Ragab et al., 2019). However, DVI should be considered a multidimensional concept with three primary dimensions: cognitive, affective, and conative (Králiková et al., 2020). The beliefs and knowledge about a particular destination belong to the cognitive part; interactions with and feelings toward a particular destination belong to the affective image; the conative dimension evolved from the cognitive and affective images and is considered to be analogous to behavior. Both cognitive and affective images are the best predictors of destination visitation (Králiková et al., 2020).

#### Local Community Participation and Destination Visit Image

The current tourism literature suggests several roles the local community could take in tourism development (Thetsane, 2019) as the participation of locals in tourism activities is occasioned by the social, economic, and environmental benefits of tourism (Stylidis et al., 2014). Tourism improves the standard of living for locals at the tourist destination, boosts economic growth, and brings direct and indirect foreign investments that affect business activities, although this may deter locals from participating in tourism activities due to its negative impacts (Li, 2002). Recently, researchers have suggested that LCP should be considered an important predictor for sustainable tourism (Thetsane, 2019) because LCP is critical in creating first-class environmental conditions for tourists and the essential constituents of the current tourism development (Chili and Ngxongo, 2017). However, few studies have focused so far on how LCP itself feels about its imposed roles (Thetsane, 2019).

According to Richards and Hall (2003), if LCP was not involved from the initial planning stage of tourism, it becomes much harder to bring it on board at a later stage. Local community participation is the central point for the continuous supply of hospitality areas, food preparation, knowledge, transport, amenities, and services for visitors (Chili and Ngxongo, 2017). Tourist satisfaction, meanwhile, is likely to be greater in destinations supported by LCP and if the local residents take pride in tourism because they understand how the destination adapts to change (Page, 2007). Eventually, local people and their communities have turned into the objects of development but not the subject of that development (Lekaota, 2015). In other words, tourists can be attracted to places where local residents are more friendly, hospitable, and honest. Thus, LCP needs to be intensely involved and given an active role in the development of tourism activities in a given destination (Michael, 2009 and should participate in the planning and managing of tourism activities within the locality (Yang et al., 2019).

Cultural heritage tourism sites are one type of destination that has been receiving increased attention (Lee et al., 2020). According to Alshboul (2016), the cultural impact associated with tourism activities is based on the relationship between locals and visitors; so the image of international visitors, the level of education, and the level of communication between residents and visitors can affect the level of LCP. Cultural heritage tourism also provides an avenue for the interchange between visitors and residents, creating a better cultural understanding that may raise awareness for preserving traditional customs and values (Gursoy et al., 2010). Thus, the lack of LCP in tourism development and the realization of tourism planning remains a serious dilemma (Moscardo, 2015), as it can assist the development of more appropriate decisions and boost local motivation, and it may play an active role in forming a suitable environment for cultural heritage visits; indeed, tourist satisfaction tends to be greater in areas where LCP encourages and values tourism (Richards and Hall, 2003). It will likely reduce the level of hostility between tourism providers, tourists, and communities through the actions taken and the consequences of those actions (Nyaupane et al., 2006). Hence, LCP must be actively involved in all tourism-related intentions (Huo, 2016) and, more importantly, local cultural heritage is a valuable asset that supports the cultural and economic values of host communities in the region visited by tourists (Yang et al., 2017). Local community participation can help protect intangible cultural heritage and preserve its uniqueness (Scherl and Edwards, 2007). Therefore, the following hypothesis is proposed:

H1a: LCP positively influences DVI.

#### Authenticity and Destination Visit Image

An increasing number of tourists have been seeking both authentic and memorable experiences at cultural heritage sites. This is because heritage tourists are more likely to be interested in destination authenticity (Girish and Lee, 2019), which is the quality of being original, genuine, and traditionally made (Nguyen and Cheung, 2016). It asserts whether an object is what it appears to be in reality (Tian et al., 2020). Authenticity is of great importance for the competitiveness of tourism destinations, and it can be assessed in terms of the originality of services and attractions at the destination, the symbolic meanings attached to attraction, and the perceptions of visitors of what makes them authentic (Wang, 1999). Thus, destination authenticity is a measure of how genuine tourists perceive the destination to be and how much they enjoy their cultural tourism experiences (Kolar and Zabkar, 2010). The perception of an authentic experience results from the interests, attitudes, behaviors, and experiences of the tourists acquired throughout their lives (Oliver, 1999). Destination authenticity not only provides tourists with in-depth destination experiences but also influences their decision-making processes (Lee et al., 2020).

Although destination authenticity has been highlighted as an important concept, little research has been conducted to explore its role in the decision-making process for visiting heritage destinations (Lee et al., 2020). Studies have been conducted to associate cultural heritage as a tourist attraction (Masoud et al., 2019), and people from around the world spend their leisure time engaging in activities within different locales that have been designed to meet the specific needs of tourists (Nguyen and Cheung, 2016). Authenticity thus obviously plays an important role in terms of whether a particular destination satisfies the DVI of visitors (and locals).

Authenticity is associated with cultural and heritage tourism and, as a concept, has been captured in heritage protection (Jamal and Hill, 2004), which asserts that the actual experience and self-recognition of an object are what attracts tourists. Authenticity is determined by active intangible cultural heritage and trends related to existentialism and constructivism (Nguyen and Cheung, 2016). Constructive authenticity aims to improve and maintain tourist destinations, while existentialism authenticity aims to attract and maintain the level of tourism (Kim and Bonn, 2016). The main motivation for tourists to travel to a destination lies in their quest for authenticity (Zhu, 2015). The concept of authenticity can be captured in-depth in the consumer-based model (Zhou et al., 2013), which demonstrated that the motivations of tourists to visit a destination are positively influenced by perceived authenticity. As a result, the following hypothesis is proposed:

H1b: Authenticity positively influences DVI.

#### Access to Local Products and Destination Visit Image

One main measurement of familiarity with a destination is the geographic distance between the origin of tourists and the destination country (Hu and Ritchie, 1993), as tourists prefer to travel to destinations culturally dissimilar from their home countries (Ju et al., 2021). According to Wang et al. (2021a), tourism is itself one of the products of a country, and the intention to visit can be regarded as similar to the intention to purchase. The term “access to local products” refers to the ease with which a product can be obtained or consumed (Kaufmann et al., 2012). Researchers have indicated that, although consumers have motives for purchasing specific things, this motivation does not translate into purchasing behaviors if it is difficult to access those products (Vermeir and Verbeke, 2004). In specific research fields (e.g., green purchasing behavior), the most essential reason consumers exhibited low consciousness, which lagged behind their actual purchasing behavior, is that they could not easily acquire information about the products or the products were not easily available (Kaufmann et al., 2012); so, consumers stated that they had low motivation to purchase those products or services.

Some studies have suggested that improving the ability to access local products significantly influences the perceptions and intentions of visitors to visit a particular destination; for example, Diao et al. (2017) indicated that the growth in transport facilities has directly promoted the degree to which Chinese cities are opening up and has strengthened their communication with the rest of the world (Xueliang, 2013). The service quality offered by travel agencies also affects the promotion of attractive tourism products (Mak et al., 2011). Tourists are guided by their preferences, choice, and behavior when looking for products to purchase (Lehto et al., 2004). Tourists also consider the shopping environment and the ambiance, variety of goods, and services of shops (Liu et al., 2020). In line with prior research, factors related to the local host influence the shopping tourism satisfaction of consumers. This should be kept in mind when considering that some remote cultural heritage sites are now easily accessible to tourists, thus, increasing the number of visitors in those locations (Zhang et al., 2018). As a result, the following hypothesis is being advanced:

H1c: Access to local products positively influences DVI.

### Destination Visit Image: Toward Tourist Satisfaction and Loyalty

The influence of tourist perception, DVI, and satisfaction has been a topic of great research interest in tourism (Rajesh, 2013). Over time, DVI piqued the curiosity of both practitioners and academics, and it has been examined in relation to the destination brand since the 1990s (Almeyda-Ibáñez and George, 2017). The image can be described as “the feelings of people of anything that they were aware” (Boulding, 1956, p. 15) and “a way of organizing the different stimuli received on a daily basis and help make sense of the world in which we live in” (Mayo, 1973). Numerous researchers have asserted that DVI is only one component of the destination brand (Konecnik and Gartner, 2007, cited in Herle, 2018). Overall, DVI can be characterized in two ways: the affective image, in which the destination image is formed based on the beliefs and feelings of tourists toward the destination; and the cognitive image, which is created as a result of the assessment of the tourists of the historical, social, political, and economic characteristics of the destinations (Wang et al., 2021a). Destination visit image is critical to the decision-making process of tourists because a destination that lacks a favorable or positive image will find it difficult to compete (Králiková et al., 2020). The images of tourists of a destination can be quite personal and subjective because tourists can form their views based on individual perceptions of the place (Alcocer and Ruiz, 2019). Therefore, DVI is not only important regarding tourist satisfaction within a given destination but also influences the behavior of tourists, including on-site experiences, evaluations, and destination loyalty (Králiková et al., 2020). Alcocer and Ruiz (2019) reported that DVI highly influenced the satisfaction of tourists toward a cultural destination, while Ragab et al. (2019) found that a positive DVI results in the higher satisfaction of tourists, further leading to positive word-of-mouth recommendation and revisit intention. Thus, the following hypotheses are presented:

H2a: DVI positively influences tourist satisfaction.

H2b: DVI positively influences tourist loyalty.

### The Satisfaction of Tourists Toward Tourist Loyalty

In the tourist literature, consumer satisfaction is an assessment made by customers based on their interactions with service providers, and it can be used to forecast future experiences (Crosby et al., 1990). In tourism, satisfaction primarily relates to the judgment of travelers concerning tourist destinations based on their on-site experience. The contentment of travelers is a favorable mental state created by the experience travelers had while traveling (Meng and Uysal, 2008), and the loyalty of tourists to a destination has a strong correlation with tourist satisfaction.

**FIGURE 1:**
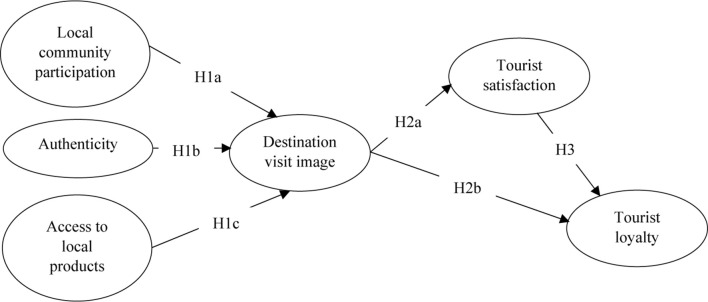
Conceptual research model.

Previous studies have found that satisfaction affects the future behavioral intentions of tourists (Petrick, 2004; Prayag and Ryan, 2012). Consumer loyalty and satisfaction are firmly and inexorably related according to both practitioners and academics (Oliver, 1999). According to Melo et al. (2017), the least satisfied tourists are less likely to return to a destination compared with their more satisfied counterparts. Ragab et al. (2019) suggested that tourist satisfaction positively and significantly influences not only the positive word-of-mouth recommendation but also the revisit intention. Those results are in line with that of Chi and Qu (2008), who found that satisfaction positively influenced destination loyalty. The following hypothesis is therefore presented, and this paper, thus, has proposed an integrative theoretical research model based on above discussion (see Figure 1):

H3: Tourist satisfaction positively influences tourist loyalty.

## Materials and Methods

### Research Approach

Using the deductive approach, a positivist philosophical paradigm was adopted for the empirical testing of hypotheses. The underlying patterns and causal relationships were identified, and the obtained results were generalized. The outcomes of the testing of the hypotheses contribute to the domain of related knowledge. The selection of positivism was to reduce bias, as the single external reality in a value-free manner was examined objectively. The exploratory nature of this study provides valuable new insights and prospects on the phenomenon under study. For the current study, a survey was conducted to obtain a large sample from a large population (Saunders et al., 2011). The survey was deemed reliable by the respondents, and the obtained data can be standardized and easily compared (Sekaran, 2006). Considering the limitations of resources, a cross-sectional research design was considered.

### Sampling and Data Collection

Kaiping Diaolou (clock towers), and the villages in which they stand, lie within the South Asian tropical climate zone in a hilly area of oriental rice cultivation. The city center of Kaiping is located on the Changjiang River, 140 km (87 miles) west of the Pearl River Delta, on the outskirts of the county of Kaiping. The construction of the Diaolou began in the 14th-15th centuries, but the peak period of its construction was in the late 19th century and the beginning of the 20th century (UNESCO World Heritage Convention, 2007); the Diaolou and the surrounding villages followed traditional cultural and land-use patterns from the turn of the 20th century, notable for the tidy traditional communities in front, with high defensive buildings for flood preservation behind the low-rise village residences, which are separated by narrow lanes, with fish ponds and ancestral halls in front and the tall Diaolou surrounded by bamboo groves behind (Huadong, 2013).

For the present study, a non-probability sampling method was considered. It is difficult for social science studies to acquire an accurate sampling frame or track down potential respondents from a population of interest to address research questions (Saunders et al., 2011). A non-probability sampling method is typically based on the subjective judgment of the researcher (Sekaran, 2006). In this study, a convenience non-probability sampling strategy was adopted. Data were gathered based on two characteristics: (1) geography—that is, domestic respondents visiting Kaiping in Guangdong; and (2) time-based—that is, respondents selected during the Tomb-Sweeping holiday in 2021. The Tomb-Sweeping holiday is a long-standing public holiday in China during which the majority of Chinese people engage in tourism activities. This facilitated data collection and ensured a more representative population. The questionnaire items were translated into Chinese using the back-translation method. A pretest with 30 respondents was undertaken to assure the use and validity of the designed instrument and to prevent any difficulties that could compromise the quality of the gathered data.

Based on Cochran’s Formula, a reasonable sample size of 384 respondents was determined (Wang et al., 2020b). Hair et al. (2010a) noted that sample sizes greater than 200 offer an adequate margin of error, although some researchers recommend that a sample size of at least 200 or between 10 and 20 cases per parameter is appropriate for using structural equation modeling (Kline, 2015; Wang and Zhang, 2021). In total, 448 questionnaires were returned; after eliminating invalid and incomplete questionnaires, 406 usable questionnaires were retained, which exceeded the value of the limited respondents. Furthermore, a latent variable was included in the confirmatory factor analysis (CFA) model by connecting it to observable factors, as Podsakoff et al. (2003) suggested that a common latent factor can examine the common method bias (CMB). The new model was evaluated by standardized regression before comparing it with the original model. Both models were found similar after the comparison. Finally, Harman’s single factor test was undertaken to identify if the CMB affected the results. The results showed an exploratory factor analysis with a single factor accounted for 48.498% of the variance, which is less than the 50% benchmark value. This indicated that CMB was not a substantial issue in this study.

### Operationalization

The research instrument adopted for this study was a self-administered questionnaire. The questionnaire had four sections. The first section included the three independent variables: LCP, authenticity, and access to local products. The three items related to LCP were adapted from Gursoy et al. (2010); the four items used to measure authenticity were adapted from Lee et al. (2020); the five items related to access to local products were adapted from Puciato (2020). The second section included DVI and tourist satisfaction. The five items used to assess DVI were adapted from Wang et al. (2021a), and the three items related to tourist satisfaction were adapted from ÇavuĢoğlu et al. (2020). The third section was used to assess the endogenous dependent variable: tourist loyalty, for which three items were adapted from ÇavuĢoğlu et al. (2020). The last section elicited relevant demographic characteristics. All of the aforementioned measurement items were evaluated using a five-point Likert scale, ranging from “strongly disagree” to “strongly agree.” The comprehensive research questionnaire is presented in Appendix A.

### Demographics

Table 1 presents the demographic information for the present study. Of the 406 respondents whose answers were used, approximately 37.4% were male, and 62.6% were female; 67% were aged between 18 and 30, 16% between 31 and 45, followed by those under 18 (11.8%), 4.7% between 46 and 60, and 2 respondents were older than 61. In terms of education, 47% of the respondents have completed a 4-year bachelor’s degree, 34.7% have completed a 3-year diploma, 6.9 and 6.7% have completed high school or a master’s and above, respectively, and 4.7% have completed middle school education. Approximately 38.2% of the respondents reported earning 1,701–3,000 CNY (Chinese Yuan) in monthly income, 20.9% of respondents earned 3,001–4,500 CNY, 15.3% earned 4,501–6,000 CNY, 15% earned below 1,700 CNY, and 10.6% of respondents earned more than 6,001 CNY.

**TABLE 1:** Sample characteristic (*N* = 406).

Items	Characteristic	Frequency	Percentage (%)
Gender	Male	152	37.4
	Female	254	62.6
Age	Below 18	48	11.8
	18–30	272	67.0
	31–45	65	16.0
	46–60	19	4.7
	Above 61	2	0.5
Income level	Below 1,700	61	15.0
	1,701–3,000	155	38.2
	3,001–4,500	85	20.9
	4,501–6,000	62	15.3
	Above 6,001	43	10.6
Education level	Middle school	19	4.7
	High school	28	6.9
	Diploma	141	34.7
	Bachelor	191	47.0
	Masters and above	27	6.7

## Data Analysis and Results

This study used IBM SPSS (version 19) (IBM, Armonk, New York, NY, United States) to perform descriptive analysis and ANOVA. This was followed by CFA and SEM using Analysis of Moment Structures (AMOS). The CFA results reflect whether the variables under study correspond to the latent variables, particularly for validating or confirming the theories explored (Suhr, 2006). The proposed hypotheses were tested using SEM, given that it works not only with a single simple or multiple linear regression but also with a system of regression equations (Wang and Wong, 2020).

### Confirmatory Factor Analysis

To analyze the reliability of the study, Peterson (1994) recommended using an index form for a Cronbach’s alpha value of 0.7 and above for the acceptable internal reliability. For the convergent validity of the measurement model, the composite reliability (CR) should be more than 0.7, and the average variance extracted (AVE) should be greater than 0.5 (Hair et al., 2010a). The results showed that validity and reliability were achieved (see Table 2).

**TABLE 2:** Construct validity and reliability.

Constrcuts (Cronbach’s Alpha)	Items	Loadings	CR	AVE
Local community participation (α = 0.955)	(LCP1) I feel at home in this community (LCP2) I have an interest in knowing what goes on in this community (LCP3) I regret moving away from this community for some reason	0.960 0.957 0.898	0.957	0.881
Authenticity (α = 0.917)	(AU1) I like the way Kaiping in the village blends with attractive landscape and historical ensemble, which offer many interesting places to visit (AU2) During my visit to Kaiping in the village, I feel the related history and traditional culture (AU3) This visit provides a wealth of traditional knowledge and cultural heritage into the historical ear (AU4) Kaiping is the village is a place where I can experience the traditional Chinese lifestyle	0.927 0.788 0.917 0.824	0.923	0.750
Access to local products (α = 0.922)	(ATLP1) My process of selecting Kaiping as a destination is based on objective premises (ATLP2) The current location of Kaiping is only one taken into account (ATLP3) The location of Kaiping is the most important factor for me to select a heritage destination (ATLP4) My decision of choosing heritage destination (i.e., Kaiping) result from some stimulating activities undertaken by local or regional authorities (ATLP5) I collect information before selecting Kaiping as a heritage destination (e.g., service quality, shop’s atmosphere, tourism gifts’ prices, etc.)	0.872 0.886 0.894 0.817 0.743	0.925	0.713
Destination visit image (α = 0.951)	(DVI1) Kaiping as a destination left a deep impression because it is a relaxing destination (DVI2) Kaiping as a destination left a deep impression because it is an arousing destination (DVI3) Kaiping as a destination left a deep impression because its natural resource (climate and landscape richness) (DVI4) Kaiping as a destination left a deep impression because its tourist infrastructure (restaurants, hotels, and accommodations) (DVI5) Kaiping as a destination left a deep impression because its culture, history, and art (gastronomy, theatre festivals, concerts, crafts, and folklore)	0.930 0.931 0.854 0.875 0.871	0.952	0.797
Tourist satisfaction (α = 0.937)	(TS1) I am happy with my decision to visit Kaiping (TS2) I am pleased to have stayed in Kaiping (TS3) I think I did the right thing visiting Kaiping.	0.925 0.900 0.914	0.937	0.833
Tourist loyalty (α = 0.877)	(TL1) I will continue my visit to cultural heritage destinations like Kaiping (TL2) I am ready to recommend Kaiping to my family and friends (TL3) Although the price of other destinations is cheaper than cultural heritage destinations, this high price in heritage destinations can be accepted	0.792 0.904 0.855	0.888	0.725

*CR, composite reliability; AVE, average variance extracted.*

The CR should be higher than the AVE. To assess the discriminate validity, the maximum shared squared variance (MSV) and the average shared squared variance (ASV) were both considered. The MSV and ASV should both be smaller than the AVE (Hair et al., 2010b). No correlation between each construct exceeded 0.9 (Wang et al., 2021b); so, the validity of the measurement model is established, as shown in Table 3.

**TABLE 3:** The correlation coefficients matrix and descriptive statistics.

No.	Research construct	1	2	3	4	5	6	AVE	MSV	ASV
1	Tourist satisfaction	***0.913***						0.833	0.486	0.237
2	Local community participation	0.448	***0.938***					0.881	0.349	0.186
2	Authenticity	0.306	0.215	***0.866***				0.750	0.426	0.214
3	Access to local products	0.503	0.317	0.653	***0.844***			0.713	0.426	0.286
4	Destination visit image	0.389	0.591	0.598	0.578	***0.893***		0.797	0.358	0.279
5	Tourist loyalty	0.697	0.485	0.381	0.564	0.451	***0.852***	0.833	0.486	0.278

*Items with bold denote square root of AVE. AVE, average variance extracted; MSV, maximum shared squared variance; ASV, averages shared squared variance.*

Next, the fit of the measurement model was checked. The model fit indices demonstrated that the measurement model was a good fit to the data (χ^2^ = 644.172, degree of freedom (df) = 211, *p* < 0.001, χ^2^/df = 3.053, root mean square residual (RMR) = 0.039, goodness of fit (GFI) = 0.874, comparative fit index (CFI) = 0.955, adjusted goodness of fit index (AGFI) = 0.835, parsimony goodness of fit index (PGFI) = 0.668, parsimony normed fit index (PNFI) = 0.779, parsimonious comparative fit index (PCFI) = 0.796, non-normed fit index (NFI) = 0.934, incremental fit index (IFI) = 0.955, tucker lewis index (TLI) = 0.946, relative fit index (RFI) = 0.821, root mean square error of approximation (RMSEA) = 0.071) and met the threshold values.

### Analysis of Structural Equations

**FIGURE 2:**
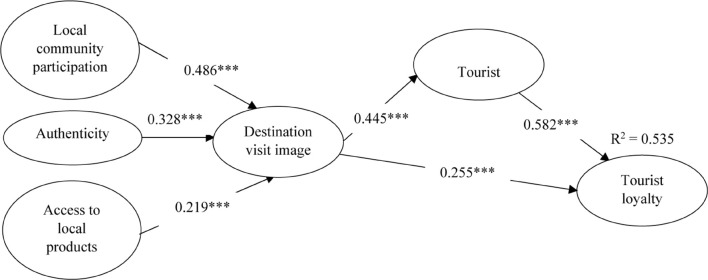
Structural model results. ***p < 0.001; Critical ratio (C.R.) > 1.96.

The following stage was to conduct SEM to validate the hypotheses of the study. The outcomes indicated that the suggested structural model possessed a sufficient level of goodness-of-fit statistics (χ^2^ = 747.801, df = 217, *p* < 0.001, χ^2^/df = 3.446, RMR = 0.082, GFI = 0.863, CFI = 0.944, AGFI = 0.826, PGFI = 0.679, PNFI = 0.792, PCFI = 0.81, NFI = 0.924, IFI = 0.945, TLI = 0.935, RFI = 0.911, RMSEA = 0.078). Figure 2 and Table 4 show the details for these findings.

**TABLE 4:** Results of the structural equation modeling.

Hypothesized paths	β	C.R.	Sig.	Decision
H1a: Local community participation → Destination visit image	0.486	12.084	***	Supported
H1b: Authenticity → Destination visit image	0.328	6.720	***	Supported
H1c: Access to local products → Destination overall image	0.219	4.373	***	Supported
H2a: Destination visit image → Tourist satisfaction	0.445	8.860	***	Supported
H2b: Destination visit image → Tourist loyalty	0.255	5.488	***	Supported
H3: Tourist satisfaction → Tourist loyalty	0.582	11.252	***	Supported

Standardized regression weights (β). C.R., critical ratio. *** Denotes the significance value below 0.001.

According to Wang et al. (2021a), a path coefficient below 0.1 indicates a small effect, a path coefficient of about 0.3 indicates a moderate effect, and a path coefficient above 0.5 indicates a large effect. The results indicate that LCP positively influenced DVI with major effects (β = 0.486, *p* < 0.001); thus, H1a was supported. Authenticity and access to local products positively and moderately influenced DVI, with authenticity influencing DVI with a path coefficient of 0.328, *p* < 0.001, and access to local products influencing DVI with a path coefficient of 0.219, *p* < 0.001. Hence, H1b and H1c were supported. The results indicate that DVI moderately positively influenced tourist satisfaction with a path coefficient of 0.445, *p* < 0.001, and tourist loyalty with a path coefficient of 0.255, *p* < 0.001. Thus, H2a and H2b were supported. Furthermore, tourist satisfaction had a major influence on tourist loyalty (β = 0.582, *p* < 0.001). Thus, H3 was supported.

## Discussion and Conclusion

Most previous studies on destination familiarity have used experiential familiarity to investigate the influence of destination familiarity on the DVI, attitude, and intentions of tourists toward a given destination. There is, however, a lack of understanding of how informational familiarity accumulates *via* indirect exposure to a destination. This study deconstructed informational familiarity as LCP, destination authenticity, and access to local products, which would influence DVI, tourist satisfaction, and tourist loyalty toward cultural heritage destinations. Local communities should be engaged in tourism activities because tourism can enhance the standard of living of community members. Nevertheless, recent studies have indicated that the role of LCP cannot only be considered in terms of tourism planning, development, and operation but also as an influence on the perceptions and satisfaction of tourists with a given destination (Richards and Hall, 2003; Gursoy et al., 2010). Chili and Ngxongo (2017) indicated that LCP is very important in the tourism industry as community members can be considered to be one of the tourism products of a destination, and their inputs in the decision-making processes for tourism development should be a focal point. Another study, by Thetsane (2019), demonstrated that LCP positively and significantly influenced the attitudes, perceptions, and decision-making processes of tourists at all levels. Our results indicate that LCP positively influenced DVI with a moderate effect size (β = 0.486, *p* < 0.001), which means that the more that local friendly hosts enthusiastically participate in tourism activities, the more comfortable tourists will be and the easier it will be for visitors to enjoy their tourism journey.

Our results indicate that destination authenticity positively and moderately influenced DVI, with a path coefficient of 0.328, *p* < 0.001. Tourists who visit cultural heritage destinations are more concerned about the reality of the place. Both constructive and existential destination authenticity significantly influenced the image of tourists regarding the destination. This is in line with the findings of Lee et al. (2020), which showed that authenticity significantly influenced the attitudes, peer influence, confidence in making decisions, and emotions of tourists, further leading to their desire and intention to visit heritage destination choices. Such outcomes correspond with the findings of other prior studies (Meng and Choi, 2016; Kim et al., 2019).

Access to local products also positively and significantly influenced the DVI of tourists, with a path coefficient of 0.219, *p* < 0.001. According to Wang et al. (2021b), there are sometimes external motivational barriers (e.g., convenience, price) to purchasing behavior that are intense and have a differential effect on the tourists. Diao et al. (2017) argued that better transport facilities positively and significantly influenced the perceptions of tourists visiting China, and Liu et al. (2020) stated that shopping environment, shop ambiance, variety of goods, and better services significantly influenced the visiting perceptions and satisfaction of tourists. In other words, the location, service quality, and available information played a significant role in shaping the decision-making processes of tourists. This is also in line with prior studies, which argued that access to products has a significant relationship with consumer purchase intention and behavior (Liu et al., 2020; Wang et al., 2021b).

In prior studies, DVI positively influenced the intention of tourists to visit a given destination (Wang et al., 2021a); according to Alcocer and Ruiz (2019), DVI has been shown to be an important predictor of positive tourist satisfaction, positive word-of-mouth recommendation, and revisit intention. Our results indicate that DVI was a significant variable with a positive and moderate relationship with tourist satisfaction (β = 0.445, *p* < 0.05) and tourist loyalty (β = 0.255, *p* < 0.05). Tourists with a positive image of a particular destination have high satisfaction with that place, which also increases the probability that they will revisit, recommend the place to others, or be willing to accept higher prices at a cultural destination compared with other destinations. Meanwhile, tourist satisfaction also positively influenced tourist loyalty with a large effect size, because there is a positive correlation between the two (β = 0.582, *p* < 0.05) for the Kaiping heritage destination. This is in line with Petrick (2004), who reported that that tourist satisfaction significantly influenced loyalty; Prayag and Ryan (2012) reported similar results.

### Theoretical Contributions

Most empirical studies have proposed destination familiarity as a single-dimension construct measured through previous destination visiting experience (Stylidis, 2020) that has a positive correlation with destination image (Chi et al., 2020). Recent studies have shown that familiarity with a tourist destination can be conceptualized as having two dimensions: experiential and informational (Chen and Lin, 2012; Kim et al., 2019). However, Kim et al. (2019) indicated that most previous studies on destination familiarity have only measured experiential familiarity and failed to consider the indirect familiarity (without consumption experience) of consumers with a destination, and this element also needs to be incorporated into the measurement of destination familiarity. Thus, this study proposed that informational familiarity can be measured through LCP, authenticity, and access to local products.

First, researchers have underestimated the role of LCP in the perceptions, satisfaction, intentions, and behaviors of tourists. According to Chili and Ngxongo (2017), community involvement can strengthen the character of most tourism destinations. However, most investigations have proposed that the role of LCP should be in tourism planning, development, and operation in either the initial or the progress stage. However, tourist satisfaction tends to be greater in areas where hosts support and take pride in tourism (Richards and Hall, 2003). The positive DVI of tourists is positively influenced by the performance of local residents, and this translates into satisfaction and loyalty toward the cultural heritage destination. The study outcomes thus offer an alternative perspective for considering the role of LCP in tourism activities.

Second, authenticity and access to local products significantly influenced the DVI of tourists for a particular cultural heritage destination. Cultural heritage sites differ from many other tourism products and services, and authenticity plays an important role in determining whether a particular cultural heritage destination can satisfy the DVI of tourists. Tourists from different parts of the world spend their leisure time visiting these remote places, which are not easily accessible. Easy access to local products significantly improves the DVI of tourists for cultural heritage destinations. Therefore, this study provides a basis for future research to replicate and clarify the motivations and perceptions of tourists, as well as their visiting behaviors regarding cultural heritage sites.

Third, research related to cultural heritage destinations has rarely been characterized for developing countries (e.g., China), and insufficient attention has been paid to how local communities influence tourist cultural heritage destinations. Our results confirmed that LCP, authenticity, and access to local products positively and significantly influenced the DVI, satisfaction, and loyalty of tourists toward cultural heritage destinations. These findings are especially significant for communities in developing countries such as China and Egypt (Ragab et al., 2019), where LCP, authenticity, and access to local products could play a significant role in marketing for cultural heritage tourism.

### Practical Implications

There are several practical consequences for cultural heritage destinations based on the results of this study. First, this study emphasized the importance of destinations developing deeper marketing strategies with LCP, because the feelings of tourists about visiting a particular heritage destination are highly influenced by LCP. Local communities cannot isolate local people from tourism planning, development, or promotion. The invisible information (i.e., informational familiarity) interchange between tourists and local people highly influences the DVI of tourists, thus, local tourism planners should re-consider establishing a role for local people in tourism activities and developing tourism activities involving local people, such as encouraging locals to engage in routine tourism activities, promoting homestays, and recruiting more locals as publicity commentators to stimulate the sense of LCP and promote local culture to visitors.

Second, because the findings of this study showed that authenticity and access to local products positively influenced DVI, thus, cultural heritage sites should ensure the accuracy of the information published on social media and websites about the destination. Visitors should receive the right information so that they can perceive that they made the right decision to visit the destination. Such web content should transfer positive information to potential visitors, such as how easy it is to visit the place, the competitive price of local products, and an atmosphere that is friendly to all visitors. Overall, cultural heritage destinations should create easy access to accurate information so that potential visitors can develop a positive DVI.

The results of this study also showed that DVI positively and significantly influenced tourist satisfaction and loyalty. Destination image can be classified into affective and cognitive images, so cultural heritage destinations operators should not only continue to preserve natural resources (e.g., climate, landscape, environment), but also pay more attention to developing better human-made resources, such as restaurants, hotels, accommodations, theatre festivals, and folklore, among others. Tourist satisfaction and loyalty toward a cultural heritage destination (i.e., Kaiping) can also be assessed in terms of the historical, social, political, and economic characteristics of a destination. Therefore, on the one hand, cultural heritage destination (e.g., Kaiping) operators should provide better services with local communities for potential tourists, while, on the other hand, they also need to develop strategies to preserve natural resources.

### Limitations

The study sample consisted of only two respondents above the age of 61 and did not reflect the total Chinese population, which means that the results may not be generalizable. This study was carried out with a very limited scope and focused on the Kaiping cultural heritage destination in Guangdong province, China. Thus, the context is only applicable to this area and may vary from other places. A sample size larger than 30 and less than 500 has been deemed appropriate for most research (Wang et al., 2020a), and this study has successfully collected 406 respondents for data analysis, but a larger sample size could provide more normal distribution and produce better outcomes (Saunders et al., 2011). This study is also one of few to evaluate the influence of LCP on the DVI and satisfaction of tourists from the decision-making process perspective of a tourist. The model should be duplicated and tested in other locations to further confirm its usefulness and validity.

## Data Availability Statement

The raw data supporting the conclusion of this article will be made available by the authors, without undue reservation.

## Ethics Statement

Ethical review and approval was not required for the study on human participants in accordance with the local legislation and institutional requirements. The patients/participants provided their written informed consent to participate in this study.

## Author Contributions

All authors listed have made a substantial, direct and intellectual contribution to the work, and approved it for publication.

## Conflict of Interest

The authors declare that the research was conducted in the absence of any commercial or financial relationships that could be construed as a potential conflict of interest.

## Publisher’s Note

All claims expressed in this article are solely those of the authors and do not necessarily represent those of their affiliated organizations, or those of the publisher, the editors and the reviewers. Any product that may be evaluated in this article, or claim that may be made by its manufacturer, is not guaranteed or endorsed by the publisher.

## Appendix

**TABLE A1:** Questionnaire Items.

Constructs	Items
Local community participation	(LCP1) I feel at home in this community (LCP2) I have an interest in knowing what goes on in this community (LCP3) I regret moving away from this community
Destination authenticity	(AU1) I like the way the village of Kaiping blends with attractive landscape and historical ensemble, which offer many interesting places to visit (AU2) During my visit to the village of Kaiping, I felt the related history and traditional culture (AU3) This visit provides a wealth of traditional knowledge and cultural heritage to experience (AU4) Kaiping is a village where I can experience the traditional Chinese lifestyle
Access to local products	(ATLP1) My process of selecting Kaiping as a destination was based on objective premises (ATLP2) The current location of Kaiping was only one taken into account (ATLP3) The location of Kaiping is the most important factor for me in selecting a heritage destination (ATLP4) My decision in choosing a heritage destination (i.e., Kaiping) was a result of some stimulating activities undertaken by local or regional authorities (ATLP5) I collected information before selecting Kaiping as a heritage destination (e.g., service quality, shop atmosphere, gift prices)
Destination visit image	(DVI1) Kaiping as a destination left a deep impression because it is a relaxing destination (DVI2) Kaiping as a destination left a deep impression because it is an arousing destination (DVI3) Kaiping as a destination left a deep impression because of its natural resource (climate and landscape richness) (DVI4) Kaiping as a destination left a deep impression because of its tourist infrastructure (restaurants, hotels, and accommodations) (DVI5) Kaiping as a destination left a deep impression because of its culture, history, and art (gastronomy, theatre festivals, concerts, crafts, and folklore)
Tourist satisfaction	(TS1) I am happy with my decision to visit Kaiping (TS2) I am pleased to have stayed in Kaiping (TS3) I think I did the right thing in visiting Kaiping
Tourist loyalty	(TL1) I will continue visiting cultural heritage destinations like Kaiping (TL2) I am ready to recommend Kaiping to my family and friends (TL3) Although the price of other destinations is cheaper than cultural heritage destinations, this high price is acceptable

*All of the questionnaire items were evaluated using a five-point Likert scale, ranging from “strongly disagree” to “strongly agree.”*
